# Cord blood acrylamide levels and birth size, and interactions with genetic variants in acrylamide-metabolising genes

**DOI:** 10.1186/s12940-021-00715-0

**Published:** 2021-04-01

**Authors:** Janneke Hogervorst, Hubert W. Vesper, Narjes Madhloum, Wilfried Gyselaers, Tim Nawrot

**Affiliations:** 1 Centre for Environmental Sciences, Hasselt University, Agoralaan gebouw D, 3590 Diepenbeek, Hasselt, Belgium; 2 Division of Laboratory Sciences, Centers for Disease Control and Prevention, Atlanta, USA; 3 Department of Obstetrics, East-Limburg Hospital, Genk, Belgium; 4 Department of Public Health & Primary Care, Leuven University, Leuven, Belgium

**Keywords:** Cord blood acrylamide, Birth weight, Birth length, Head circumference, SNPs in acrylamide-metabolising genes

## Abstract

**Background:**

Up to now, 3 epidemiological studies have shown clear inverse associations between prenatal acrylamide exposure and birth size. In addition to studying the association between acrylamide and birth size, we investigated the interaction between acrylamide and polymorphisms in acrylamide-metabolising genes, with the aim of probing the causality of the inverse relationship between acrylamide and fetal growth.

**Methods:**

We investigated the association between prenatal acrylamide exposure (acrylamide and glycidamide hemoglobin adduct levels (AA-Hb and GA-Hb) in cord blood) and birth weight, length and head circumference in 443 newborns of the ENVIR*ON*AGE (ENVIRonmental influence *ON* AGEing in early life) birth cohort. In addition, we studied interaction with single nucleotide polymorphisms (SNPs) in *CYP2E1*, *EPHX1* and *GSTP1,* using multiple linear regression analysis.

**Results:**

Among all neonates, the body weight, length and head circumference of neonates in the highest quartile was − 101 g (95% CI: − 208, 7; p for trend = 0.12), − 0.13 cm (95% CI: − 0.62, 0.36; p for trend = 0.69) and − 0.41 cm (− 0.80, − 0.01; p for trend = 0.06) lower, respectively, compared to neonates in the lowest quartile of AA-Hb in cord blood, For GA-Hb, the corresponding effect estimates were − 222 g (95% CI: − 337, − 108; p for trend = 0.001), − 0.85 (95% CI: − 1.38, − 0.33; p for trend = 0.02) and − 0.55 (95% CI: − 0.98, − 0.11; p for trend = 0.01), respectively. The associations for GA-Hb were similar or stronger in newborns of non-smoking mothers. There was no statistically significant interaction between acrylamide exposure and the studied genetic variations but there was a trend of stronger inverse associations with birth weight and head circumference among newborns with homozygous wildtypes alleles for the *CYP2E1* SNPS and with variant alleles for a *GSTP1* SNP (rs1138272).

**Conclusions:**

Prenatal dietary acrylamide exposure, specifically in the form of its metabolite glycidamide, was inversely associated with birth weight, length and head circumference. The interaction pattern with SNPs in *CYP2E1,* although not statistically significant, is an indication for the causality of this association. Other studies are needed to corroborate this finding.

## Background

Acrylamide is a food processing-generated compound present in various heat-treated carbohydrate-rich foods, such as cookies, potato chips, French fries and coffee, due to preparation of the foods at high temperatures. It is classified as a probable human carcinogen by the International Agency for Research on Cancer [1]. In addition to carcinogenicity, acrylamide has been shown to cause neurotoxicity in animals and occupationally exposed humans, and reproductive and developmental toxicity in animals [2].

Lately, evidence has accumulated that acrylamide exposure during pregnancy may have an adverse effect on fetal growth. In rodents, a decrease in offspring body weight due to gestational acrylamide exposure has been observed but only at doses that caused overt toxicity to the mothers [2]. Those doses are not reached through human dietary intake [2]. In humans, acrylamide and its metabolite glycidamide readily pass the placental barrier, and a moderate correlation between cord and maternal blood was observed, 0.48 (range: 0.27.- 0.86) and 0.38 (range: 0.20–0.73) for acrylamide and glycidamide, respectively [3]. The authors of this study deducted that the in vivo dose in fetal and maternal blood is about the same and that the placenta gives negligible protection of the fetus to exposure from acrylamide and glycidamide [3]. To date, three epidemiological studies have shown that higher gestational acrylamide exposure is linked to reduced fetal growth [4–6]. In light of these findings, the European Food Safety Authority (EFSA) in its 2015 acrylamide risk assessment called for more epidemiological research on the association between acrylamide intake and birth outcome.

Through the present study, we aim to contribute more evidence on the causality of the association between prenatal acrylamide exposure and birth outcomes by assessing the link between acrylamide exposure biomarkers and birth weight, birth length and birth head circumference, and studying the potential modifying role of single nucleotide polymorphisms (SNPs) in acrylamide-metabolising genes (*CYP2E1*, *EPHX1* and *GSTP)* in this association. CYP2E1 is a phase-I biotransformation enzyme with a major role in the metabolism of acrylamide to glycidamide, the compound that is hypothesised to be responsible for the carcinogenic effect of acrylamide [2]. EPHX1 too is a phase-I biotransformation enzyme that metabolises highly reactive epoxides that have mutagenic and carcinogenic potential to less reactive diols and in that way they often lead to the detoxification of compounds [7]. With regard to acrylamide’s metabolite glycidamide, EPHX1 will metabolise this compound to the less toxic glyceramide [2]. GSTP1 is a member of the glutathione-S-transferase enzyme family that play an important role in detoxification by conjugating reduced glutathione to many hydrophobic and electrophilic compounds. In this way, it detoxifies many compounds, among which acrylamide and glycidamide. Considering the important role of the mentioned enzymes in the detoxification of acrylamide and glycidamide, we hypothesise that newborns differ in their susceptibility to the adverse effect of acrylamide on their growth in utero, based on their genotype of the mentioned genes. Thus, we expect to see differences in the strength of the associations between acrylamide and glycidamide and birth size parameters between the strata of genotypes of these enzymes.

## Methods

### Study population and data collection

We recruited mother-newborn pairs in the East-Limburg Hospital in Belgium, between Friday 12 p.m. and Monday 7 a.m. from February 2nd 2010 until May 18th 2013. The catchment area of the hospital included the province Limburg in Belgium and combines both urban and suburban to rural areas with population densities of the municipalities ranging from 82 to 743 inhabitants/km^2^. Participants were recruited if the mother was able to fill out a questionnaire in Dutch. The overall participation rate of eligible mothers was 61%. The ENVIR*ON*AGE birth cohort was representative of all births in Flanders with regard to maternal age and education, parity, new-born’s sex, ethnicity, and birth weight.

Upon arrival in the hospital for delivery, participating mothers gave written informed consent and completed questionnaires on among other age, pre-gestational body mass index (BMI), maternal education, occupation, smoking status, alcohol consumption, use of medication, parity and new-born’s ethnicity in the postnatal ward after delivery.

Perinatal parameters, such as new-born’s sex, birth date, birth weight and length, gestational age and Apgar score, were collected after birth from the medical records. We included only full-term (≥36 weeks) and singleton pregnancies. All included new-borns were healthy and free of anomalies confirmed by both prenatal ultrasound examination and postnatal assessment immediately by paediatricians. Further details of the study are reported elsewhere [8]. The analysis described in this paper involved 443 mother-child pairs for which we had acrylamide hemoglobin adduct measurements in umbilical cord blood and complete covariable data. At the time we selected the mother-child pairs for acrylamide exposure assessment, there were 1387 mother-newborn pairs included into the study. The inclusion for this cohort is still ongoing and there are now more than 2000 mother-newborn pairs included. Selection for acrylamide exposure assessment was based on whether the children had already visited our lab for the follow-up visit at 4 years of age (we preferentially included those newborns), on missing data for possible covariables (we chose newborns with the least missing data) and on the amount and quality of the cord blood samples.

#### Acrylamide and glycidamide hemoglobin adducts in cord blood

EDTA cord blood samples (*n* = 500, among which were 25 duplicate samples) were sent to the Centers for Disease Control and Prevention (CDC) Protein Biomarker Laboratory (Atlanta, USA) to measure acrylamide (AA-Hb) and glycidamide (GA-Hb) hemoglobin adducts. Details of the methodology can be found elsewhere [9, 10]. Briefly, 300 μL of whole cord blood, of which plasma and buffy coat layers formed after centrifugation were removed, was analysed using an optimised Edman reaction and HPLC/tandem mass spectrometry (HPLC/MS/MS). Hemoglobin adducts of acrylamide and glycidamide with the N-terminal valine of the hemoglobin protein chains were measured as N-[2-carbamoylethyl]valine-pentafluorophenylhydantion (PFPTH) derivative and N-[2-hydroxycarbamoyl-ethyl]valine-pentafluorophenylhydantion (PFPTH) derivative, respectively. Concentrations of AA-Hb and GA-Hb were reported relative to the amount of hemoglobin (pmol per g of Hb). and total hemoglobin was measured as cyanmethemoglobin, which is formed from methemoglobin by reaction with cyanide. The light absorption of the resulting red colored complex was measured spectrophotometrically at 540 nm.The lower limits of detection for this method are 3 pmol/g of Hb for acrylamide and 4 pmol/g of Hb for glycidamide.

#### Single nucleotide polymorphisms (SNPs) in acrylamide-metabolising genes

In the context of the ENVIR*ON*AGE birth cohort, genotyping data of a set of candidate SNPs (*n* = 210) in genes related to several domains of health (cognition, obesity, cardiovascular disease, ageing) were available. From this dataset, we selected three SNPs in *cytochrome P450 2E1 (CYP2E1)*, namely rs2480258, rs915906 and rs11101888 (the latter as a proxy for rs6413432), one SNP in *epoxide hydrolase 1* (*EPHX1)* (rs1051740) and two in *glutathione-s-transferase P1 (GSTP1)* (rs1695 and rs1138272); all are genes involved in acrylamide metabolism.

Genomic DNA was isolated from placental tissue from the fetal side of the placenta using the QIAamp® DNA Mini Kit (Qiagen Inc., Venlo, The Netherlands), according to the manufacturer’s instructions. In short, cells were lysed by using AL buffer and RNA and proteins were inactivated by RNAse A and proteinase K, respectively. To purify DNA, ethanol was added to the samples before applying them to the QIAamp Mini spin columns. Wash buffers and an elution buffer were used to ultimately collect the DNA from the samples. The concentration and purity of the isolated DNA were evaluated using the NanoDrop® ND-1000 spectrophotometer (Thermo Scientific, Wilmington, DE, USA). Genotyping was conducted using the Biotrove OpenArray SNP Genotyping Platform at the Harvard Medical School-Partners Healthcare Center for Genetics and Genomics. For the current analysis, we only included participants with a sample genotyping call rate ≥ 90% across all of the 210 SNPs in the analyses.

### Statistical analysis

We performed multiple linear regression analysis to assess the association between cord blood acrylamide and glycidamide hemoglobin adducts and birth weight, birth length and birth head circumference. Covariables in the models were pre-gestational BMI, the number of cigarettes smoked during pregnancy, maternal weight gain during pregnancy, parity, gestational age, date of delivery and newborn’s sex. In a sensitivity analysis, passive smoking of the mother, vegetable, fruit and fish intakes and consumption of soda drinks were included in the models as covariables. In another sensitivity analysis, we added the ratio of glycidamide to acrylamide hemoglobin adducts to the model, as a marker of the degree of the metabolic conversion of acrylamide to glycidamide in the body, to see whether the associations observed, if any, were perhaps due to this metabolic efficacy rather than to acrylamide or glycidamide.

We performed subgroup analyses for newborns of mothers who did not smoke during pregnancy. Furthermore, we analysed whether maternal smoking or new-born’s sex modified the association between acrylamide exposure and birth outcomes.

Multiplicative interaction between acrylamide and SNPs was tested using product terms of the continuous acrylamide variable and the categorical genotype variable. For this analysis, we considered acrylamide hemoglobin adducts as a biomarker of exposure. We did not analyse the interaction between genotypes and glycidamide adducts because this marker is a representation of internal acrylamide exposure after metabolism, which is influenced by the studied genotypes. To show genotype strata-specific associations between acrylamide and birth outcomes, we used a dominant genetic model for all SNPs (homozygous wildtype versus heterozygous or homozygous variant). We calculated sum scores for *CYP2E1* and *GSTP1* by summing up variant allele numbers for the 3 SNPs in *CYP2E1* and the 2 SNPs in *GSTP1*, respectively. For the *CYP2E1* and *GSTP1* sum scores, we dichotomised the scores into 0 versus 1 or more variant alleles.

Lastly, we analysed the association between the SNPs in acrylamide-metabolising genes and the ratio of glycidamide to acrylamide hemoglobin adducts; the latter, as a marker of the degree of conversion of acrylamide to glycidamide in the body, is possibly influenced by the these SNPs.

Q-Q plots of the residuals were inspected in order to check the assumptions of linear models.

We used SAS software (version 9.4; SAS Institute Inc., Cary, NC, USA) for all statistical analyses.

## Results

Based on the 25 samples that were analysed in duplicate, intraclass correlation coefficients (ICC) were 0.97 for acrylamide and 0.98 for glycidamide. For glycidamide, 1 sample was below the LOQ, and 57 samples were non-reportable due to an unacceptable signal-to-noise ratio in the instrument signal, and thus 417 out of the 475 samples had a result for glycidamide. The median acrylamide hemoglobin adduct level in the 443 newborns that we had complete data for (including covariables data) was 13.2 (range: 5.38–140.0) pmol/ g of hemoglobin. The median glycidamide hemoglobin adduct level was 13.3 (range: 5.13–144.0) pmol/ g of hemoglobin. In newborns of non-smoking mothers (*n* = 383) the median acrylamide hemoglobin adduct level was 12.5 (IQR: 10.2–15.6) and the median glycidamide hemoglobin adduct level was 12.2 (IQR: 9.8–15.6) pmol/ g of hemoglobin. Other characteristics of the study population are summarised in Table 1. The study population consisted of 229 (51.7%) boys and 214 (49.3%) girls. The median birth weight, birth length and head circumference of the newborns were 3430 (IQR: 3140–3720) grams, 50 (IQR: 49–52) centimeters and 34 (IQR: 33–35) centimeters, respectively. Several variables were associated with birth outcomes (results not shown). Boys had on average a higher birth weight, birth length and birth head circumference. The duration of the pregnancy was positively associated with all 3 birth outcomes, as was the weight gain of the mother during pregnancy. There were 63 women that smoked during pregnancy. Cigarette smoking was inversely associated with birth weight and birth length, while parity was positively associated with birth weight and head circumference. The BMI of the mother before pregnancy was only associated with birth weight.

**Table 1:** Characteristics of mother-newborn pairs (*n* = 443)

Characteristics	Median (IQR) or n (%)
**Maternal**	
Age, y	30 (27–32)
Pre-gestational BMI, kg/m^2^	23.5 (21.3–26.6)
Gestational weight gain, kg	14 (11.0–17.5)
Maternal education	
Low	48 (10.9%)
Middle	142 (32.3%)
High	250 (56.8%)
Smoking during pregnancy	
Yes	63 (14.2%)
No	380 (85.8%)
Number of cigarettes smoked per day^a^	9 (5–10)
Parity	
1	236 (53.3%)
2	152 (34.3%)
≥ 3	55 (12.4%)
**Newborn**	
Sex	
Male	229 (51.7%)
Female	214 (49.3%)
Ethnicity	
European	393 (88.7%)
Non-European	50 (11.3%)
Acrylamide hemoglobin adducts (pmol/ g hemoglobin)	13.2 (10.4–17.6)
Glycidamide hemoglobin adducts (pmol/ g hemoglobin) (*n* = 393)	13.3 (10.2–18.0)
Glycidamide to acrylamide ratio (*n* = 393) Gestational age, w	0.92 (0.76–1.10) 40 (39–40)
Birth weight, g	3430 (3140–3720)
Birth height, cm	50 (49–52)
Birth head circumference, cm	34 (33–35)

*IQR* interquartile range

^a^among the women who reported to have smoked during pregnancy

The frequencies of the genotypes and the corresponding *p*-value for Hardy-Weinberg equilibrium are shown in Table 2. None of the 6 SNPs showed a statistically significant deviation from Hardy-Weinberg equilibrium.

**Table 2:** Genotype frequencies

SNP ID	Homozygous wildtype n (%)	Heterozygous n (%)	Homozygous variant allele n (%)	Hardy-Weinberg ***p*** value
rs915906, *CYP2E1*	196 (70)	78 (28)	6 (2)	0.59
rs2480258, *CYP2E1*	174 (62)	99 (35)	9 (3)	0.26
rs11101888, *CYP2E1*	234 (83)	47 (17)	1 (0)	0.40
rs1051740, *EPHX1*	157 (54)	107 (37)	25 (9)	0.27
rs1695, *GSTP1*	125 (43)	140 (48)	26 (9)	0.13
rs1138272, *GSTP1*	234 (82)	50 (17)	2 (1)	0.70

None of the SNPs by itself showed a statistically significant association with birth outcomes (Table 3).

**Table 3:** Associations between genotypes of acrylamide-metabolising enzymes and birth outcomes

SNP^**a**^	Estimated change in birth outcome (95% CI)					
	*Birth weight (gram)*	*p*	*Birth length (cm)*	*p*	*Birth head circumference (CM)*	*p*
rs915906	24.5 (−36.9, 86.0)	0.43	−0.17 (− 0.44, 0.10)	0.22	− 0.04 (− 0.26, 0.19)	0.74
rs2480258	19.1 (−40.3, 78.5)	0.53	− 0.19 (− 0.45, 0.07)	0.16	− 0.02 (− 0.24, 0.20)	0.85
rs11101888	18.8 (− 56.0, 93.7)	0.62	0.05 (− 0.28, 0.38)	0.77	0.19 (− 0.07, 0.44)	0.15
rs1051740	−17.6 (−76.3, 41.1)	0.56	0.05 (− 0.21, 0.31)	0.70	− 0.02 (− 0.23, 0.20)	0.89
rs1695	2.0 (−56.2, 60.2)	0.95	−0.02 (− 0.28, 0.23)	0.86	0.04 (− 0.17, 0.25)	0.71
rs1138272	−7.3 (−86.6, 72.1)	0.86	−0.09 (− 0.44, 0.26)	0.60	− 0.03 (− 0.32, 0.27)	0.86

Adjusted for: maternal pre-pregnancy BMI (kg/m2), maternal weight gain during pregnancy, maternal smoking during pregnancy (n cigarettes/day), parity (n children), gestational age (weeks), newborn’s sex and date of delivery

^a^modeled is the regression coefficient of 1 or 2 variant alleles of the SNP compared to the homozygous wildtype

In unadjusted analyses among all newborns, cord blood acrylamide hemoglobin adduct levels showed a statistically significant inverse association with birth weight, length and head circumference when acrylamide adduct levels were modelled as a continuous variable but there was only a significant linear trend over the quartiles of acrylamide adducts for head circumference (Table 4). In newborns of women who did not smoke during pregnancy, acrylamide adduct levels were inversely associated with birth weight and head circumference when acrylamide was modelled as a continuous variable but there was no clear linear trend over the quartiles (Table 4).

**Table 4:** Associations between acrylamide and glycidamide hemoglobin adducts in cord blood and birth outcomes

		Unadjusted regression coefficient (95% CI)							Multivariable-adjusted regression coefficient (95% CI)						
		Continuous exposure variable (per 10 pmol/g hemoglobin)		Quartiles of exposure					Continuous exposure variable (per 10 pmol/g hemoglobin)		Quartiles of exposure				
	n	Regression coefficient (95% CI)^a^	p	Q1 REF	Q2	Q3	Q4	*p* for trend	Regression coefficient (95% CI)^a^	p	Q1 REF	Q2	Q3	Q4	*p* for trend
***Acrylamide hemoglobin adducts among all participants***															
Weight, g	443	−59 (−88, −31)	< 0.001	1	−74 (−188, 41)	−98 (− 212, 17)	− 149 (− 263, 34)	0.08	−40 (− 71, −9)	0.01	1	−30 (− 127, 67)	−102 (−200, − 5)	−101 (−208, 7)	0.12
Length, cm	442	−0.24 (−0.37, − 0.11)	< 0.001	1	− 0.22 (− 0.73, 0.30)	0.17 (− 0.35, 0.68)	−0.35 (− 0.86, 0.16)	0.19	−0.17 (− 0.31, − 0.03)	0.02	1	−0.03 (− 0.48, 0.41)	0.15 (−-0.29, 0.60)	−0.13 (− 0.62, 0.36)	0.69
Head circumference, cm	432	−0.15 (− 0.24, − 0.05)	0.003	1	−0.33 (− 0.72, 0.06)	−0.51 (− 0.90, − 0.12)	−0.54 (− 0.92, − 0.15)	0.03	−0.13 (− 0.24, − 0.01)	0.03	1	−0.16 (− 0.52, 0.19)	−0.45 (− 0.81, − 0.09)	−0.41 (− 0.80, − 0.01)	0.06
***Acrylamide hemoglobin adducts among neonates from non-smoking mothers***															
Weight, g	380	−58 (−113, − 4)	0.04	1	−67 (− 181, 47)	− 88 (− 202, 25)	− 30 (− 170, 110)	0.45	−52 (− 98, − 5)	0.03	1	−26 (− 123, 72)	− 101 (− 200, − 2)	− 77 (− 197, 43)	0.19
Length, cm	379	−0.17 (− 0.42, 0.08)	0.18	1	− 0.21 (− 0.73, 0.31)	0.19 (− 0.33, 0.70)	0.27 (− 0.37, 0.90)	0.35	−0.18 (− 0.40, 0.04)	0.10	1	− 0.04 (− 0.49, 0.41)	0.15 (− 0.31, 0.60)	−0.00 (− 0.56, 0.55)	0.86
Head circumference, cm	369	−0.23 (− 0.42, − 0.04)	0.02	1	−0.32 (− 0.72, 0.07)	−0.48 (− 0.87, − 0.08)	−0.36 (− 0.85, 0.13)	0.12	−0.22 (− 0.40, − 0.05)	0.01	1	−0.16 (− 0.52, 0.21)	−0.42 (− 0.80, − 0.05)	−0.48 (− 0.94, − 0.03)	0.07
***Glycidamide hemoglobin adducts among all participants***															
Weight, g	393	−80 (− 117, − 44)	< 0.001	1	− 161 (− 282, − 40)	− 175 (− 297, − 54)	−267 (− 387, − 146)	< 0.001	− 53 (− 90, − 16)	0.005	1	− 144 (− 246, − 43)	− 154 (− 258, − 49)	−222 (− 337, − 108)	0.001
Length, cm	392	− 0.35 (− 0.52, − 0.19)	< 0.001	1	−0.55 (− 1.09, − 0.01)	− 0.59 (− 1.13, − 0.05)	−1.10 (− 1.64, − 0.56)	0.001	− 0.24 (− 0.41, − 0.08)		1	−0.46 (− 0.93, 0.01)	−0.48 (− 0.96, − 0.00)	−0.85 (− 1.38, − 0.33)	0.02
Head circumference, cm	382	− 0.17 (− 0.30, − 0.05)	0.007	1	− 0.69 (− 1.10, − 0.27)	− 0.74 (− 1.16, − 0.33)	− 0.74 (− 1.15, − 0.32)	< 0.001	− 0.11 (− 0.25, 0.03)		1	− 0.61 (− 0.99, − 0.23)	− 0.61 (− 1.00, − 0.21)	− 0.55 (− 0.98, − 0.11)	0.01
***Glycidamide hemoglobin adducts among neonates from non-smoking mothers***															
Weight, g	331	−121 (− 193, − 50)	< 0.001	1	− 164 (− 285, − 43)	− 176 (− 296, − 50)	−176 (− 327, − 25)	0.01	− 104 (− 165, − 43)	0.001	1	−152 (− 255, − 50)	−161 (− 268, − 54)	−225 (− 355, − 95)	0.002
Length, cm	330	− 0.48 (− 0.80, − 0.15)	0.004	1	−0.56 (− 1.11, − 0.01)	−0.61 (− 1.17, − 0.05)	−0.64 (− 1.33, 0.05)	0.10	− 0.42 (− 0.70, − 0.13)	0.004	1	−0.50 (− 0.98, − 0.02)	− 0.56 (− 1.06, − 0.06)	−0.81 (− 1.42, − 0.21)	0.04
Head circumference, cm	320	− 0.36 (− 0.62, − 0.11)	0.005	1	−0.69 (− 1.11, − 0.27)	−0.72 (− 1.15, − 0.29)	−0.55 (− 1.09, − 0.02)	0.003	− 0.31 (− 0.55, − 0.07)	0.01	1	−0.61 (− 1.01, − 0.22)	−0.61 (− 1.02, − 0.19)	−0.63 (− 1.14, − 0.13)	0.007

Adjusted for: maternal pre-pregnancy BMI (kg/m^2^), maternal weight gain during pregnancy, maternal smoking during pregnancy (n cigarettes/day), parity (n children), gestational age (weeks), newborn’s sex and date of delivery

^a^Regression coefficient (95% confidence interval) per 10 pmol/g hemoglobin increase in AA-Hb and GA-Hb adducts

In multivariable-adjusted models among all newborns, acrylamide adduct levels were still inversely associated with all 3 birth outcomes with regression coefficients of − 40 (95% CI: − 71, − 9; p: 0.01) grams, − 0.17 (95% CI: − 0.31, − 0.03; p: 0.02) centimeters, and − 0.13 (− 0.24, − 0.01; p: 0.03) centimeters per increment of 10 pmol per gram of hemoglobin for birth weight, length and head circumference, respectively, but the effect estimates were slightly reduced compared to the unadjusted estimates. Among neonates of non-smoking mothers, the effect estimate for birth weight was slightly weaker than for the unadjusted analysis but the effect estimates for birth length and head circumference were virtually unchanged (Table 4).

The associations with glycidamide adducts are stronger and show clearer dose-response relationships than those with acrylamide. In addition, glycidamide adducts show a clear inverse association with birth length, in contrast to what was observed for acrylamide adducts. In unadjusted analyses among all newborns and among newborns of non-smoking mothers, glycidamide was inversely associated with all 3 birth outcomes. All of the associations showed a clear dose-response across the quartiles of glycidamide adducts except for head circumference among newborns of non-smoking mothers. In multivariable-adjusted analyses, the regression coefficients of a 10 pmol per gram hemoglobin increase in glycidamide adducts were − 53 (95% CI: − 90, − 16; p: 0.005) grams, − 0.24 (95% CI: − 0.41, − 0.08; p: 0.004) centimeters and − 0.11 (95% CI: − 0.25, 0.03; p: 0.11) centimeters for birth weight, length and head circumference, respectively. In newborns of women who did not smoke during pregnancy, the regression coefficients of the continuous glycidamide adduct level variable were roughly twice as large as those for the whole group.

For acrylamide, but particularly for glycidamide, the effect estimates were larger in newborns of mothers who did not smoke during pregnancy (Table 4). The interaction with smoking was not significant for birth weight (p interaction = 0.50), birth length (p interaction = 0.88) or head circumference (p interaction = 0.25) for acrylamide and birth length for glycidamide (p interaction = 0.15), but (borderline) significant for birth weight (p interaction = 0.04) and head circumference (p interaction = 0.07) for glycidamide. Newborn’s sex was not a statistically significant effect modifier of the association between acrylamide hemoglobin adduct levels and birth weight (p interaction = 0.36) birth length (p interaction = 0.35), or head circumference (p interaction = 0.55) for acrylamide and the corresponding *p* values were 0.19, 0.34 and 0.39 for glycidamide.

The effect estimates of acrylamide and glycidamide did not change profoundly when we additionally adjusted for passive smoking of the mother, consumption of vegetables, fruits, fish and soda drinks (results not shown). When we adjusted for the ratio between glycidamide and acrylamide hemoglobin adduct levels, the effect estimates for glycidamide hemoglobin adducts remained virtually the same.

**Fig. 1:**
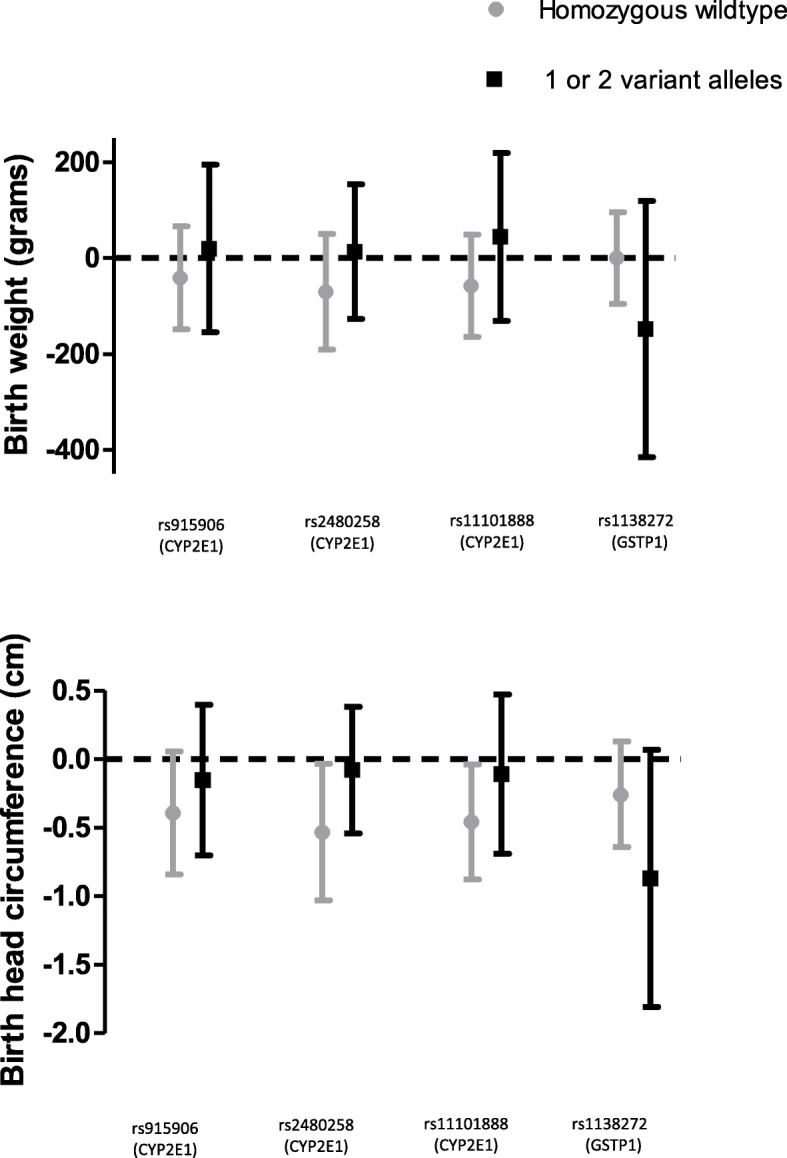
The association between acrylamide hemoglobin adducts and birth weight and head circumference in newborns from non-smoking mothers according to selected genotypes

There was no statistically significant interaction between any of the genetic variables and acrylamide (Table 5). Some differences between genotypes are, however, worth mentioning. Among neonates of non-smoking mothers, there was only an inverse association between acrylamide and birth weight in those who were homozygous wild types for rs915906, rs2480258 and rs11101888 in *CYP2E1*. When the three CYP2E1 SNPs were summed, only neonates with no variant alleles showed a clear inverse association between acrylamide hemoglobin adducts and birth outcomes, both among all neonates and among those born to non-smoking mothers. Furthermore, only neonates from non-smoking mothers with at least 1 variant allele of rs1138272 in *GSTP1* had an inverse association between acrylamide and birth weight. With regard to birth head circumference, similar differences were observed for the same genetic variants. The sum of the 2 *GSTP1* SNPs did not modify the association between acrylamide and birth outcomes. The above-mentioned differences between genotypes are shown in Fig. 1, for birth weight and head circumference.

**Table 5:** Interactions between genetic variants and cord blood acrylamide adduct levels on birth outcomes

	All				Non-smoking mothers			
	n	Acrylamide regression coefficient	95% CI	*p* for interaction	n	Acrylamide regression coefficient	95% CI	*p* for interaction
***Birth weight***								
*CYP2E1* rs915906 = 0^a^	196	−62.1	− 130.3, 6.1	0.80	169	−37.0	− 145.0, 71.0	0.87
*CYP2E1* rs915906 = 1^a^	83	−54.9	− 143.4, 33.6		70	12.3	−167.4, 192.0	
*CYP2E1* rs2480258 = 0	174	−74.5	− 146.3, − 2.6	0.64	148	−66.9	− 187.8, 54.4	0.59
*CYP2E1* rs2480258 = 1	107	−45.1	− 121.9, 31.8		92	7.8	− 135.3, 150.9	
*CYP2E1* rs11101888 = 0	234	−77.8	−136.4, − 19.3	0.17	199	−57.9	−164.2, 48.4	0.34
*CYP2E1* rs11101888 = 1	47	10.2	−154.4, 174.7		41	35.3	−141.1, 211.7	
*EPHX1* rs1051740 = 0	157	−55.6	−141.8, 30.5	0.15	137	−83.1	−249.6, 83.3	0.49
*EPHX1* rs1051740 = 1	131	−43.9	−112.6, 24.8		109	7.8	−103.3, 118.9	
*GSTP1* rs1695 = 0	129	−51.4	120.4, 17.6	0.85	105	−34.4	−146.8, 78.0	0.82
*GSTP1* rs1695 = 1	166	−68.3	− 162.2, 5.6		144	−42.4	−196.2, 111.4	
*GSTP1* rs1138272 = 0	237	−51.7	−109.2, 5.8	0.46	202	−0.1	−95.9, 95.7	0.28
*GSTP1* rs1138272 = 1	52	− 121.5	− 347.0, 104.1		43	− 148.4	− 415.6, 118.8	
Sum of 3 *CYP2E1* SNPs = 0	163	− 78.0	− 149.9, −6.0	0.38	138	−77.8	− 200.5, 45.0	0.35
Sum of 3 *CYP2E1* SNPs ≥1	103	−36.3	−118.8, 46.2		88	44.4	−103.2, 191.9	
Sum of 2 *GSTP1* SNPs = 0	117	−42.5	−114.5, 29.5	0.59	99	−24.2	−137.9, 89.5	0.84
Sum of 2 *GSTP1* SNPs ≥1	166	−85.4	− 168.9, −1.9		145	−63.7	−212.1, 84.7	
***Birth length***								
CYP2E1 rs915906 = 0	196	− 0.21	− 0.50, 0.09	0.69	169	−0.09	− 0.56, 0.39	0.42
CYP2E1 rs915906 = 1	83	−0.19	− 0.61, 0.24		70	0.21	−0.65, 1.06	
CYP2E1 rs2480258 = 0	174	−0.22	−0.52, 0.09	0.76	148	−0.14	−0.66, 0.37	0.52
CYP2E1 rs2480258 = 1	107	− 0.19	− 0.56, 0.17		92	0.07	−0.63, 0.78	
CYP2E1 rs11101888 = 0	234	− 0.22	− 0.48, 0.04	0.32	199	− 0.02	−0.49, 0.46	0.97
CYP2E1 rs11101888 = 1	47	− 0.23	−0.91, 0.46		41	−0.12	−0.92, 0.67	
EPHX1 rs1051740 = 0	157	− 0.23	− 0.61, 0.16	0.50	138	0.28	−0.45, 1.01	0.85
EPHX1 rs1051740 = 1	131	−0.11	− 0.43, 0.20		109	0.02	−0.50, 0.54	
GSTP1 rs1695 = 0	129	−0.27	−0.58, 0.04	0.33	105	−0.28	−0.80, 0.24	0.19
GSTP1 rs1695 = 1	166	−0.04	−0.40, 0.32		144	0.55	− 0.11, 1.21	
GSTP1 rs1138272 = 0	237	−0.11	−0.35, 0.14	0.46	202	0.11	−0.31, 0.53	0.93
GSTP1 rs1138272 = 1	52	−0.81	−1.89, 0.27		43	−0.28	−1.75, 1.18	
Sum of 3 CYP2E1 SNPs = 0	167	−0.24	−0.54, 0.07	0.64	138	−0.16	−0.68, 0.36	0.55
Sum of 3 CYP2E1 SNPs ≥1	105	−0.21	−0.60, 0.17		88	−0.00	−0.71, 0.70	
Sum of 2 GSTP1 SNPs = 0	120	−0.22	−0.55, 0.12	0.59	99	−0.24	−0.77, 0.29	0.33
Sum of 2 GSTP1 SNPs ≥1	167	−0.10	−0.47, 0.27		145	0.44	−0.21, 1.09	
***Birth head circumference***								
CYP2E1 rs915906 = 0	192	−0.10	−0.38, 0.17	0.42	165	−0.38	−0.84, 0.07	0.56
CYP2E1 rs915906 = 1	81	−0.19	−0.47, 0.08		68	−0.14	−0.71, 0.43	
CYP2E1 rs2480258 = 0	171	−0.15	−0.44, 0.14	0.19	145	−0.53	−1.03, − 0.03	0.22
CYP2E1 rs2480258 = 1	104	−0.16	− 0.40, 0.08		89	−0.07	− 0.54, 0.40	
CYP2E1 rs11101888 = 0	229	−0.22	− 0.44, 0.01	0.36	194	−0.46	− 0.88, − 0.04	0.31
CYP2E1 rs11101888 = 1	47	−0.21	− 0.71, 0.29		40	−0.13	− 0.72, 0.46	
EPHX1 rs1051740 = 0	157	−0.23	− 0.51, 0.06	0.54	137	−0.39	− 0.92, 0.15	0.91
EPHX1 rs1051740 = 1	125	−0.09	− 0.40, 0.22		103	−0.33	− 0.86, 0.20	
GSTP1 rs1695 = 0	121	−0.13	− 0.43, 0.17	0.31	101	−0.29	− 0.83, 0.25	0.84
GSTP1 rs1695 = 1	163	−0.23	− 0.50, 0.05		142	−0.46	− 0.96, 0.04	
GSTP1 rs1138272 = 0	228	−0.16	− 0.38, 0.06	0.39	197	−0.26	− 0.64, 0.13	0.34
GSTP1 rs1138272 = 1	51	−0.44	−1.20, 0.33		42	−0.87	−1.81, 0.07	
Sum of 3 CYP2E1 SNPs = 0	160	−0.16	−0.46, 0.14	0.24	135	−0.58	−1.01, − 0.06	0.15
Sum of 3 CYP2E1 SNPs ≥1	100	−0.16	−0.41, 0.10		85	0.02	−0.46, 0.51	
Sum of 2 GSTP1 SNPs = 0	113	−0.07	−0.40, 0.26	0.70	95	−0.27	−0.82, 0.29	0.59
Sum of 2 GSTP1 SNPs ≥1	164	−0.27	−0.54, 0.00		143	−0.49	−0.96, − 0.01	

Adjusted for: maternal pre-pregnancy BMI (kg/m^2^), maternal weight gain during pregnancy, maternal smoking during pregnancy (n cigarettes/day), parity (n children), gestational age (weeks), newborn’s sex and date of delivery

^a^0 = homozygous wildtype, 1 = 1 or 2 variant alleles for SNPs

There were no associations between any of the SNPs and the ratio of glycidamide to acrylamide hemoglobin adducts (Table 6).

**Table 6:** Associations between SNPs in acrylamide-metabolising genes and the ratio of glycidamide to acrylamide hemoglobin adducts

SNP ID	n	Homozygous wildtype	n	Heterozygous	*p*	n	Homozygous variant allele	*p*
		Regression coefficient (95% CI)		Regression coefficient (95% CI)			Regression coefficient (95% CI)	
**All**								
rs915906, *CYP2E1*	196	Ref	78	0.004 (−0.07, 0.08)	0.91	6	−0.12 (− 0.37, 0.14)	0.37
rs2480258, *CYP2E1*	174	Ref	99	0.04 (−0.03, 0.10)	0.30	9	−0.06 (− 0.26, 0.14)	0.56
rs11101888, *CYP2E1*	234	Ref	47	−0.04 (− 0.13, 0.05)	0.35	1	0.83 (0.34, 1.32)	0.001
rs1051740, *EPHX1*	157	Ref	107	−0.05 (− 0.12, 0.01)	0.10	25	−0.05 (− 0.16, 0.06)	0.36
rs1695, *GSTP1*	125	Ref	140	−0.04 (− 0.11, 0.02)	0.19	26	0.01 (−0.12, 0.13)	0.93
rs1138272, *GSTP1*	234	Ref	50	−0.01 (− 0.10, 0.07)	0.77	2	0.20 (−0.15, 0.55)	0.27
**Non-smokers**								
rs915906, *CYP2E1*	169	Ref	64	0.03 (−0.06, 0.11)	0.56	6	−0.11 (− 0.38, 0.16)	0.43
rs2480258, *CYP2E1*	148	Ref	84	0.05 (−0.03, 0.12)	0.21	8	−0.11 (− 0.34, 0.13)	0.38
rs11101888, *CYP2E1*	199	Ref	40	−0.04 (− 0.14, 0.07)	0.48	1	0.84 (0.32, 1.36)	0.002
rs1051740, *EPHX1*	137	Ref	87	−0.07 (− 0.14, 0.01)	0.08	22	−0.05 (− 0.17, 0.07)	0.39
rs1695, *GSTP1*	105	Ref	121	−0.05 (− 0.12, 0.03)	0.20	23	0.02 (−0.12, 0.16)	0.76
rs1138272, *GSTP1*	202	Ref	41	−0.02 (− 0.12, 0.08)	0.69	2	0.14 (−0.16, 0.44)	0.36

Adjusted for: maternal pre-pregnancy BMI (kg/m^2^), maternal weight gain during pregnancy, maternal smoking during pregnancy (n cigarettes/day), parity (n children), gestational age (weeks), newborn’s sex and date of delivery

## Discussion

We observed an inverse multivariable-adjusted association between acrylamide hemoglobin adducts and birth weight and birth head circumference. The association was similar in newborns of mothers who did not smoke during pregnancy.

There was a stronger association between acrylamide’s metabolite glycidamide and birth weight and head circumference and glycidamide was additionally inversely associated with birth length. The inverse association between glycidamide and birth outcomes was stronger among newborns of non-smoking mothers, particularly for birth weight and head circumference.

Although with no statistically significant interaction between acrylamide and genotype, the inverse association between acrylamide exposure and birthweight and birth head circumference was stronger in children with a homozygous wildtype genotype for the studied SNPs in *CYP2E1* and children with at least one variant allele in rs1138272 in *GSTP1,* specifically among newborns from non-smoking mothers.

The order of magnitude of the effect size of the association between gestational acrylamide exposure and fetal growth in our study is similar to that of maternal smoking. For instance, the effect estimate in the highest quartile of glycidamide adducts (− 225 g for birth weight, − 0.81 for length and − 0.63 for head circumference in neonates from non-smoking mothers) is similar to the effect size of maternal smoking in our study (− 219 g for birth weight, − 0.97 for length and − 0.45 for head circumference).

Up to now, three other studies have investigated the association between acrylamide hemoglobin adduct levels and birth outcomes, among which was one study that also used acrylamide and glycidamide hemoglobin adducts as a biomarker of exposure. All three studies observed an inverse association between gestational acrylamide exposure and indicators of fetal growth [4–6]. The consistency between all four studies that have investigated the association thus far, and between the studies using food frequency questionnaire data and biomarkers, is rather remarkable. All the three other studies adjusted largely for the same covariables that we adjusted for in this study.

Out of the three studies, including the current study, that investigated the link with birth head circumference [5, 6], ours is the second study to observe an inverse association between prenatal acrylamide exposure and head circumference.

We did not observe statistically significant interaction between acrylamide and genetic variants in genes involved in acrylamide metabolism. However, there were clear differences in the strength of the association between acrylamide and birth weight and head circumference between genotypes of *CYP2E1* SNPs and a *GSTP1* SNP (rs1138272) in newborns of non-smoking mothers. CYP2E1 has several exogenous substrates among which are N-nitrosodimethylamine, acetaminophen and alcohol. Endogenously, CYP2E1 is involved in among other arachidonic acid and prostaglandin metabolism [11]. This enzyme is highly expressed in the liver and has also been shown to be catalytically active in the fetal liver [11]. The results for the interaction with *CYP2E1* SNPs suggest that glycidamide may be more important with regard to fetal growth than acrylamide because the wildtype allele of the studied SNPs is thought to have a higher enzymatic activity than the variant allele [12–14]. A stronger effect of glycidamide than of acrylamide is reflected by the observation that the associations between glycidamide and birth outcomes in the current study were stronger than the associations with acrylamide. Strikingly, analyses on acrylamide and endometrial and ovarian cancer risks in a prospective cohort study showed that non-smoking women who were homozygous for the wild type *CYP2E1* alleles (of rs2480258, rs915906 and rs11101888) had higher risks of both endometrial and ovarian cancer than women with variant alleles for those three SNPs [15]. This pattern is similar to what we saw in the current study, where newborns of non-smoking mothers who were homozygous wildtypes for the same three SNPs showed stronger associations with birth weight and head circumference than newborns with variant alleles for these SNPs.

GSTP1 is an important phase II enzyme that protects against oxidative stress and it is involved of the metabolism of acrylamide, generating mercapturic acid derivatives of acrylamide and glycidamide that are excreted with the urine. This enzyme is primarily expressed in lung, breast bladder, and placenta [16]. Glutathione-S-transferases are present in high amounts in embryonic and fetal tissues and GSTP1 was found to be the most important GST isoform in these tissues [16]. The variant allele of the rs1138272 SNP probably leads to a reduced enzymatic activity of GSTP1 [17]. Therefore, it was expected that newborns with variant alleles of rs1138272 would show a stronger association between acrylamide and fetal growth.

We did not observe clear differences between the studied genotypes with regard to the glycidamide to acrylamide hemoglobin adduct ratio in cord blood. *CYP2E1* gene transcription is induced or inhibited by numerous factors, such as liver function, fasting, obesity, alcohol intake, medication and various dietary factors [18]. In addition, post-transcriptional processes have an important influence on CYP2E1 enzyme activity. A more subtle effect of the genotype may be masked by those other factors and processes that influence enzyme activity [18]. Nevertheless, Pelle et al. found that carriers of the variant allele (T allele) of rs2480258 in *CYP2E1* had a decreased enzyme activity, with a reduced *CYP2E1* expression phenotype at the mRNA, protein, and CYP2E1 enzyme activity level [12]. In a separate study, they observed a decreased glycidamide to acrylamide ratio in homozygous carriers of the T allele [19]. Duale et al. did not observe differences in the ratio of glycidamide to acrylamide for two SNPs in *CYP2E1* (rs6413419 and rs2515641); rs6413419 is a missense mutation. In addition, they did not observe a difference in the ratio of glycidamide to acrylamide for rs1051740 in *EPHX1,* similar to our study [7]. Also similar to our study, Doroshyenko et al. did not observe a difference in the ratio of glycidamide to acrylamide among genotypes of rs1695 and rs1138272 in *GSTP1* in human volunteers but the sample size of their study was very small (*n* = 16) [20]. In a study on 51 persons occupationally exposed to acrylamide, Huang et al. observed no influence on the glycidamide to acrylamide ratio of variant alleles of the CYP2E1*5 SNP (rs2031920/rs3813867) nor of variant alleles of the rs1051740 SNP in *EPHX1* [21].

The interactions between acrylamide and glycidamide and genotypes in the current study were not statistically significant because of the limited sample sizes, and the genotypes were not associated with the ratio of the acrylamide to glycidamide hemoglobin adducts in this study. Although the results of the acrylamide-gene interaction analyses have to therefore be interpreted cautiously, they do point towards a role of CYP2E1 and GSTP1 in the association between gestational acrylamide exposure and fetal growth and thus give some suggestion that the association could be causal.

Currently, it is only possible to speculate about the possible mechanism or mechanisms behind acrylamide’s possible adverse effect on fetal growth. Both acrylamide and glycidamide can bind to thiol groups in proteins and when those thiol groups are in important positions, the function of the protein may be impaired [22]. However, it is unknown what proteins could be involved in acrylamide’s putative adverse influence on fetal growth. Acrylamide has been shown to be associated with reduced serum insulin levels in both rats [23] and human adults [24]. Insulin is a growth factor that plays an important role in fetal growth. Acrylamide was also shown to decrease levels of plasma thyroid hormones in rats [25, 26], and among Taiwanese adolescents, there was an inverse association between levels of a urinary acrylamide metabolite and serum free thyroxin (T4) [27]. Thyroid hormones are also involved in fetal growth and development. Acrylamide may impair the function of enzymes involved in insulin and thyroid hormone regulation.

Our study has some limitations. We cannot exclude the possibility of residual confounding by factors that are associated with both acrylamide and glycidamide hemoglobin adduct levels and birth outcomes, such as another dietary exposure or a generally less heathy diet. However, when we additionally adjusted the analyses for variables that can be thought of as proxies for a healthy or unhealthy diet (consumption of vegetables, fruits, fish and soda drinks), the associations were virtually unchanged. In addition, associations were found in different study populations in which the dietary sources of acrylamide differ and were still present after adjustment for confounders and after exclusion of children from smoking mothers. Therefore, it is not likely that other factors could fully account for the observed associations. In addition, the possible effect modification by SNPs in *CYP2E1* eliminates a long list of potential confounding exposures that are not influenced by metabolism by CYP2E1.

In addition, we may have had too limited statistical power to observe statistically significant differences in the association between acrylamide hemoglobin adduct levels and birth outcomes in subgroups of genotypes. In the acrylamide-gene interaction analyses, we used the acrylamide hemoglobin adduct level as the exposure variable, while acknowledging that this introduces some random error because AA-Hb is influenced not only by the dietary intake of acrylamide but also by the genotype of acrylamide-metabolising genes. It would be interesting to see whether studies using food frequency questionnaires would observe the same indications for acrylamide-gene interactions as we did in the current study, because these studies model the external exposure and thus do not suffer from this problem. However, food frequency questionnaires come with their own major problem which is a reduced precision to estimate acrylamide exposure.

When we adjusted for the ratio between glycidamide and acrylamide hemoglobin adduct levels, the effect estimates for acrylamide and glycidamide hemoglobin adducts were virtually unchanged. From this, it can be concluded that the associations shown between acrylamide and glycidamide and birth outcomes are not due to acrylamide and glycidamide hemoglobin adduct levels merely being a proxy for the ratio of glycidamide to acrylamide, a marker for the efficiency of acrylamide to glycidamide metabolism.

Our study also has specific strengths. Our findings are generalizable as our study population is representative for the gestational segment of the population at large [8]. Another advantage of our study was the use of acrylamide biomarkers. Biomarkers should not be assumed to always be superior to other methods of acrylamide exposure assessment, such as food frequency questionnaires, because a biomarker may not represent the exposure during the relevant period for disease etiology well, e.g., due to intra-individual variations of exposure in time. However, the hemoglobin adduct biomarker may well be superior in the case of a study on fetal growth because the hemoglobin adducts of acrylamide and glycidamide represent the exposure during the last 4 months of pregnancy. This part of the pregnancy is the period in which most growth takes place. The effects sizes in the study of Pedersen et al.^6^ and in the current study, which are the two studies that have used acrylamide biomarkers, are quite comparable but they are larger than the effect sizes in the studies that used food frequency questionnaires to estimate acrylamide intake. This may be due to the greater precision of the acrylamide exposure estimate that is reached by using the biomarker. Interestingly, the two biomarker studies were also the studies that observed an inverse association with birth head circumference, whereas the study that used a food frequency questionnaire did not [5].

Our study strengthens the body of evidence that acrylamide intake at current dietary levels may have developmental effects. According to the Developmental Origins of Health and Disease hypothesis, suboptimal prenatal development likely predisposes to inferior health throughout life [28]. Reduced fetal growth has been associated with among other increased incidences of diabetes mellitus (type 2), obesity and cardiovascular disease [29]. Birth head circumference is an indication of fetal brain growth, and several studies have shown associations between decreased birth head circumference and decreased cognitive skills in childhood, even among the children within the normal range of birth size [30]. It has also been shown to be associated with an increased risk of attention deficit hyperactivity disorder (ADHD) [31]. The association between acrylamide exposure and reduced birth head circumference suggests that prenatal acrylamide exposure may have cognitive effects in later life. This study suggests that reducing acrylamide exposure during pregnancy can be beneficial for child development.

Further studies on the association between acrylamide hemoglobin adduct levels and birth outcomes in newborns, and the interaction with *CYP2E1* and *GSTP1* SNPs are needed to better understand the potential effects of acrylamide on fetal growth. Furthermore, more epidemiological research is needed on possible mechanisms of action underlying the association between prenatal acrylamide exposure and fetal development. In addition, the potential long-term effects related to acrylamide exposure in the womb should be investigated. We strongly encourage specifically epidemiological studies on this topic because the animal data suggested that no developmental effects on humans would occur at dietary doses [2].

## Conclusion

In conclusion, in utero exposure to acrylamide and especially its metabolite glycidamide are associated with decreases in birth weight, birth length and birth head circumference. Preventative measures leading to reduced exposure of pregnant women to acrylamide should be considered.

## Data Availability

The datasets used and/or analysed during the current study are available from the corresponding author on reasonable request.

## References

[1] IARC (International Agency for Research on Cancer) some industrial chemicals. IARC Monogr Eval Carcinog Risk Hum. 1994;60:1–560. PMC7681540 7869568

[2] EFSA CONTAM (2015). Panel (EFSA panel on contaminants in the food chain). Scientific opinion on acrylamide in food. EFSA J.

[3] von Stedingk H, Vikstrom AC, Rydberg P (2011). Analysis of hemoglobin adducts from acrylamide, glycidamide, and ethylene oxide in paired mother/cord blood samples from Denmark. Chem Res Toxicol.

[4] Duarte-Salles T, von Stedingk H, Granum B, Gützkow KB, Rydberg P, Törnqvist M, Mendez MA, Brunborg G, Brantsæter AL, Meltzer HM, Alexander J, Haugen M (2013). Dietary acrylamide intake during pregnancy and fetal growth-results from the Norwegian mother and child cohort study (MoBa). Environ Health Perspect.

[5] Kadawathagedara M, Tong ACH, Heude B, Forhan A, Charles MA, Sirot V, Botton J, The EDEN mother-child cohort study group. Dietary acrylamide intake during pregnancy and anthropometry at birth in the French EDEN mother-child cohort study. Environ Res. A2016;149:189–96. 10.1016/j.envres.2016.05.019. 10.1016/j.envres.2016.05.019 27208470

[6] Pedersen M, von Stedingk H, Botsivali M, Agramunt S, Alexander J, Brunborg G, Chatzi L, Fleming S, Fthenou E, Granum B, Gutzkow KB, Hardie LJ, Knudsen LE, Kyrtopoulos SA, Mendez MA, Merlo DF, Nielsen JK, Rydberg P, Segerbäck D, Sunyer J, Wright J, Törnqvist M, Kleinjans JC, Kogevinas M, the NewGeneris Consortium (2012). Birth weight, head circumference, and prenatal exposure to acrylamide from maternal diet: the European prospective mother-child study (NewGeneris). Environ Health Perspect.

[7] Duale N, Bjellaas T, Alexander J, Becher G, Haugen M, Paulsen JE, Frandsen H, Olesen PT, Brunborg G (2009). Biomarkers of human exposure to acrylamide and relation to polymorphisms in metabolizing genes. Toxicol Sci.

[8] Janssen BG, Madhloum N, Gyselaers W (2017). Cohort Profile: The ENVIRonmental influence ON early AGEing (ENVIRONAGE): a birth cohort study. Int J Epidemiol.

[9] Ospina M, Vesper HW, Licea-Perez H, Meyers T, Mi L, Myers G (2005). LC/MS/MS method for the analysis of acrylamide and glycidamide hemoglobin adducts. Adv Exp Med Biol.

[10] Vesper HW, Slimani N, Hallmans G (2008). Cross-sectional study on acrylamide hemoglobin adducts in subpopulations from the European Prospective Investigation into Cancer and Nutrition (EPIC) Study. J Agric Food Chem.

[11] Carpenter SP, Lasker JM, Raucy JL (1996). Expression, induction, and catalytic activity of the ethanol-inducible cytochrome P450 (CYP2E1) in human fetal liver and hepatocytes. Mol Pharmacol.

[12] Pelle L, Cipollini M, Tremmel R (2016). Association between CYP2E1 polymorphisms and risk of differentiated thyroid carcinoma. Arch Toxicol.

[13] Su XL, Bin B, Cui HW, Ran MR (2011). Cytochrome P450 2E1 RsaI/PstI and DraI polymorphisms are risk factors for lung Cancer in Mongolian and Han population in Inner Mongolia. Chin J Cancer Res.

[14] Verlaan M, Te Morsche RH, Roelofs HM (2004). Genetic polymorphisms in alcohol-metabolizing enzymes and chronic pancreatitis. Alcohol Alcohol.

[15] Hogervorst JG, van den Brandt PA, Godschalk RW, van Schooten FJ, Schouten LJ (2016). The influence of single nucleotide polymorphisms on the association between dietary acrylamide intake and endometrial cancer risk. Sci Rep.

[16] Raijmakers MT, Steegers EA, Peters WH (2001). Glutathione S-transferases and thiol concentrations in embryonic and early fetal tissues. Hum Reprod.

[17] Li D, Dandara C, Parker MI (2010). The 341C/T polymorphism in the GSTP1 gene is associated with increased risk of oesophageal cancer. BMC Genet.

[18] Walker K, Hattis D, Abel Russ A, Gary Ginsberg G. Physiologically-based toxicokinetic modeling for acrylamide—risk implications of polymorphisms and developmental changes in selected metabolic enzymes. Report from the George Perkins Marsh Institute, Clark University, and the Connecticut Department of Public Health to the U.S. Environmental Protection Agency under Cooperative Agreement. #827195–0. 2004.

[19] Pelle L, Carlsson H, Cipollini M (2018). The polymorphism rs2480258 within CYP2E1 is associated with different rates of acrylamide metabolism in vivo in humans. Arch Toxicol.

[20] Doroshyenko O, Fuhr U, Kunz D, Frank D, Kinzig M, Jetter A, Reith Y, Lazar A, Taubert D, Kirchheiner J, Baum M, Eisenbrand G, Berger FI, Bertow D, Berkessel A, Sörgel F, Schömig E, Tomalik-Scharte D (2009). In vivo role of cytochrome P450 2E1 and glutathione-S-transferase activity for acrylamide toxicokinetics in humans. Cancer Epidemiol Biomark Prev.

[21] Huang YF, Chiang SY, Liou SH (2012). The modifying effect of CYP2E1, GST, and mEH genotypes on the formation of hemoglobin adducts of acrylamide and glycidamide in workers exposed to acrylamide. Toxicol Lett.

[22] Friedman M (2003). Chemistry, biochemistry, and safety of acrylamide. A review. J Agric Food Chem.

[23] Totani N, Yawata M, Ojiri Y, Fujioka Y (2007). Effects of trace acrylamide intake in Wistar rats. J Oleo Sci.

[24] Lin CY, Lin YC, Kuo HK, Hwang JJ, Lin JL, Chen PC, Lin LY (2009). Association among acrylamide, blood insulin, and insulin resistance in adults. Diabetes Care.

[25] Hamdy SM, Bakeer HM, Eskander EF, Sayed ON (2012). Effect of acrylamide on some hormones and endocrine tissues in male rats. Hum Exp Toxicol.

[26] Shuming C, Jilin F, Xichun Z (2009). The moderating role of dark soy sauce to acrylamide-induced oxidative stress and neurophysiological perturbations in rats. Toxicol Mech Methods.

[27] Lin CY, Lin LY, Chen YC, Wen LL, Chien KL, Sung FC, Chen PC, Su TC (2015). Association between measurements of thyroid function and the acrylamide metabolite N-acetyl-S-(propionamide)-cysteine in adolescents and young adults. Environ Res.

[28] Aris IM, Fleisch AF, Oken E (2018). Developmental origins of disease: emerging prenatal risk factors and future disease risk. Curr Epidemiol Rep.

[29] Fernandez-Twinn DS, Ozanne SE (2006). Mechanisms by which poor early growth programs type-2 diabetes, obesity and the metabolic syndrome. Physiol Behav.

[30] Dekhtyar S, Wang H-X, Scott K, Goodman A, Koupil I, Herlitz A (2015). Associations of head circumference at birth with early-life school performance and later-life occupational prestige. Longitud Life Course Stud.

[31] Aagaard K, Bach CC, Henriksen TB, Larsen RT, Matthiesen NB (2018). Head circumference at birth and childhood developmental disorders in a nationwide cohort in Denmark. Paediatr Perinat Epidemiol.

